# Palmitoleic acid (16:1n7) increases oxygen consumption, fatty acid oxidation and ATP content in white adipocytes

**DOI:** 10.1186/s12944-018-0710-z

**Published:** 2018-03-20

**Authors:** Maysa M. Cruz, Andressa B. Lopes, Amanda R. Crisma, Roberta C. C. de Sá, Wilson M. T. Kuwabara, Rui Curi, Paula B. M. de Andrade, Maria I. C. Alonso-Vale

**Affiliations:** 1 Department of Biological Sciences, Institute of Environmental Sciences, Chemical and Pharmaceutical, Federal University of São Paulo, 210, Sao Nicolau St, Diadema, 09913-030 Brazil; 2 Department of Physiology and Biophysics, Institute of Biomedical Sciences, University of São Paulo, São Paulo, Brazil; 3 Interdisciplinary Postgraduate Program in Health Sciences, Institute of Physical Activity Sciences and Sports, Cruzeiro do Sul University, São Paulo, Brazil; 4 Department of Nursing , Health Sciences Center, Federal University of Espírito Santo, Vitória, Brazil

**Keywords:** Lipogenesis, Lipolysis, Triglyceride/fatty acid cycle, Bioenergetics, Mitochondria, Beta-oxidation

## Abstract

**Background:**

We have recently demonstrated that palmitoleic acid (16:1n7) increases lipolysis, glucose uptake and glucose utilization for energy production in white adipose cells. In the present study, we tested the hypothesis that palmitoleic acid modulates bioenergetic activity in white adipocytes.

**Methods:**

For this, 3 T3-L1 pre-adipocytes were differentiated into mature adipocytes in the presence (or absence) of palmitic (16:0) or palmitoleic (16:1n7) acid at 100 or 200 μM. The following parameters were evaluated: lipolysis, lipogenesis, fatty acid (FA) oxidation, ATP content, oxygen consumption, mitochondrial mass, citrate synthase activity and protein content of mitochondrial oxidative phosphorylation (OXPHOS) complexes.

**Results:**

Treatment with 16:1n7 during 9 days raised basal and isoproterenol-stimulated lipolysis, FA incorporation into triacylglycerol (TAG), FA oxidation, oxygen consumption, protein expression of subunits representing OXPHOS complex II, III, and V and intracellular ATP content. These effects were not observed in adipocytes treated with 16:0.

**Conclusions:**

Palmitoleic acid, by concerted action on lipolysis, FA esterification, mitochondrial FA oxidation, oxygen consumption and ATP content, does enhance white adipocyte energy expenditure and may act as local hormone.

## Background

White adipose tissue (WAT) stores triacylglycerol (TAG) and its metabolic feature involves lipogenesis and lipolysis, which are associated with changes in the volume of mature adipocytes. The activities of these two metabolic pathways vary with the need to incorporate or release fatty acids (FA), which depends on the nutritional status of the individuals, energy expenditure, hormone levels (e.g. insulin), activities of the enzymes involved in these processes (e.g. ATGL and HSL, FAS and G6PDH) and the heterogeneity existing among white adipose tissue depots [1–3].

Mitochondria play an important role in cellular function, not only as a major site of ATP production, but also by regulating energy expenditure, apoptosis signaling and production of reactive oxygen species [4]. The impact of mitochondria function and dysfunction on pre-adipocyte and adipocyte metabolism has attracted a growing body of interest over the last decade [5–7]. There are several reports linking mitochondrial dysfunction with impaired WAT energetic metabolism and the development of obesity, insulin resistance and type 2 diabetes mellitus (T2DM) [6, 8, 9]. Mitochondria are the main site for aerobic glucose and fatty acid oxidation, oxygen consumption, and generation of reactive oxygen species and ATP, which are associated with enhanced basal metabolic rate [10, 11]. Therefore, mitochondria function in WAT might play a key role to control basal metabolic rate and should be considered as a target to be investigated for the development of alternative therapies to treat/prevent obesity and related metabolic disorders.

Among several types of lipids produced and released by adipocytes, palmitoleic acid, a ω-7 monounsaturated fatty acid (16:1n7, n7) synthesized by the desaturation of palmitic acid (16:0) by stearoyl-CoA desaturase 1 (SCD1) activity, has been shown to act systemically in peripheral tissues modulating important metabolic processes. Cao and cols [12] showed that palmitoleic acid improves insulin resistance in skeletal muscle and liver and prevents hepatosteatosis. As palmitoleic acid is produced and secreted by WAT, Cao and cols., 2008 [12] named it “lipokine”. Palmitoleic acid treatment leads to increased glucose uptake, AKT phosphorylation and raises GLUT1 and GLUT4 protein levels in plasma membrane of skeletal muscle cells [13, 14]. Moreover, palmitoleic acid treatment also enhances AKT, insulin receptor, insulin receptor substrate-1 and 2 protein phosphorylation in the liver [12, 15] and exerts cytoprotective effects in pancreatic β-cells [16, 17].

We investigated herein the effects of palmitoleic acid on WAT bioenergetics. Recently, our group has shown that palmitoleic acid treatment increases adipocyte lipolysis and the content of the major lipases (ATGL and HSL) through a PPARα-dependent mechanism [18]. Besides, this lipokine enhanced glucose uptake and GLUT4 content associated with AMPK activation in these cells, favoring cellular glucose utilization towards energy production [19]. Our hypothesis is that palmitoleic acid increases white adipocyte basal metabolism. To test this proposition, 3 T3-L1 adipocytes were treated with palmitoleic acid (100 μM) for 9 days. The results were compared with those obtained with palmitic acid and vehicle under similar experimental protocol. Lipolysis, FA incorporation into lipids and FA conversion into CO_2_, as well as parameters of mitochondrial bioenergetics (fatty acid oxidation, oxygen consumption and ATP content) and proteic analysis of subunits representing OXPHOS complex (I, II, III, IV e V) were investigated. Treatment with palmitoleic acid did enhance mitochondrial activity as indicated by enhanced ATP generation, proteic expression of II, III, and V complexes of the mitochondrial electron transport chain, FA oxidation and oxygen consumption. Taken together, boosted mitochondrial bioenergetics in combination with raised lipolysis and FA reesterification leads to increased adipocyte energy expenditure via TAG/FA cycle stimulation (futile cycle). Therefore, palmitoleic acid plays a relevant role in WAT metabolism and should be considered as a candidate to be tested in obesity-related therapies.

## Methods

### Cell culture

3 T3-L1 preadipocytes were cultured in Dulbeccos Modified Eagle Medium (DMEM) containing 10% calf serum and 1% penicillin-streptomycin until confluence. Differentiation was induced 2 days post-confluence by addition of dexamethasone (1 μM), isobutylmethylxanthine (0.5 mM), insulin (1.67 μM), and 10% fetal bovine serum (FBS). After 48 h, medium was replaced by DMEM containing 10% FBS and 0.41 μM insulin [20]. Cells were cultivated for 9 days in the presence of palmitic acid (16:0), palmitoleic acid (16:1n7), each at 100 μM dissolved in ethanol 0.05% (vehicle), starting from the first day of differentiation (day 0). This dose of palmitoleic acid was found to have no cytotoxic or deleterious effects on cells as evaluated by plasma membrane integrity and DNA fragmentation during differentiation until the day 9 [21]. As we demonstrated before (18) that the palmitoleic acid effects in adipocytes are structure specific, here, we did not add more additional control such as oleic acid (18:1n9) to the cell cultures. In some experiments, etomoxir (Sigma-Aldrich), an inhibitor of CPT-1 activity, was used to inhibit fatty acid oxidation at a concentration of 40 μM, added 24 h before the experiments. Cell culture medium was changed every 2 days. All reagents and drugs were purchased from Sigma Chemical Company (St. Louis, MO, USA). Similar procedure was used in our previous studies [18].

### Lipolysis measurement

Lipolysis was estimated as the rate of glycerol (Free Glycerol Determination Kit, Sigma) and free fatty acid (FFA) (NEFA Kit RH series, Wako Diagnostics, CA, USA) released from differentiated 3 T3-L1 cells after 9-day treatment with the fatty acids or vehicle (in some experiments with or without etomoxir) during 30 min of incubation. Similar procedure was used in our previous studies [18, 22]. Results were expressed as nanomoles of glycerol per 10^6^ cells and μEq/L of free FA per well for glycerol and FFA, respectively.

### Incorporation of [1-^14^C]-palmitate into triacylglycerol

KRH (Krebs Ringer Hepes bicarbonate) buffer, pH 7.4, containing 1% BSA and 2 mM glucose plus palmitate (200 μM), saturated with a gas mixture of 95% O_2_ and 5% CO_2,_ was added to 3 T3-L1 cells after 9-day treatment with FA or vehicle. [1-^14^C]-Palmitate was then added to the buffer (1850 Bq/tube or well) and left for 2 h at 37 °C. Cells were then washed three times with phosphate buffered saline (PBS) and Dole’s reagent containing isopropanol:n-heptane:H_2_SO_4_ (4:1:0.25 vol/vol/vol) was added to the remaining reaction mixture for lipid extraction.

The mixture was transferred to polypropylene tubes, which were vortexed three times during the following 30 min. After adding n-heptane (1.5 mL) and distilled water (1.5 mL), tubes were vortexed and the mixture was decanted for additional 5 min. The upper phase was collected (in duplicates) and transferred to a scintillation vial for determination of radioactivity trapped into TAG using a β-counter (1450 LSC, Counter MicroBeta, Trilux; PerkinElmer). Results were expressed as nanomoles of FA per 10^6^ cells. Similar procedure has been used in our previous studies [18].

### Decarboxylation of [1-^14^C]-palmitate (fatty acid oxidation)

Differentiated 3 T3-L1 cells (after 9-day treatment with FA or vehicle) were incubated in KRH buffer (pH 7.4) containing BSA (1%) and [1-^14^C]-palmitate (50 μM, 1850 Bq/tube or well), saturated with a gas mixture of 95% O_2_ and 5% CO_2_, for 2 h, at 37 °C. Prior to 2 h incubation period, each well was covered with a piece of Whatman filter paper and the plate was sealed with parafilm to maintain the atmosphere saturated with the gas mixture. Following the 2 h incubation, the filter paper was soaked with 0.1 mL of ethanolamine to trap the CO2 produced, and 0.2 mL of 8 N H_2_SO_4_ was injected into the wells with the aid of a needle to rupture the cells. After 45 min of CO_2_ trapping, the filter paper was removed and transferred to scintillation vials for radioactivity counting [23, 24]. Results were expressed as nanomoles of oxidized [1-^14^C]-palmitate per 10^6^ cells.

### Oxygen consumption

Oxygen consumption rates in intact cells were measured as an indication of mitochondrial respiratory activity. After 9-day treatment with palmitic acid (100 μM), palmitoleic acid (100 μM) or vehicle, 3 T3-L1 cells were gently tripsinised, re-suspended in KRH (pH 7.4) containing BSA (0.1%), and transferred to the oxygraph (OROBOROS Oxygraph-2 k). The oxygraph chambers were previously equilibrated with KRH containing BSA 0.1% at 37 °C. Carbonyl cyanide m-chlorophenyl hydrazine (CCCP, 1 μM f.c.) was added as positive control for maximal respiratory rate (uncoupling) determination. Oxygen consumption rates were normalized by cell number and expressed as % of the control [25]. Oxygen consumption was also measured in mature 3 T3-L1 adipocytes (9 days after differentiation) treated for 24 h with palmitoleic acid (200 μM) or vehicle.

### ATP content determination

ATP content was determined in 3 T3-L1 cell lysates after 9 days of treatment with FA or vehicle (in the presence or absence of etomoxir) using an ATP bioluminescence assay kit (Roche, Mannheim, Germany). Measurements were performed in a luminometer (Biotech, model: Synergy HT). Results were normalized by cell number and expressed as percentage of control [26].

### Western blot analysis

After treatment with FA or vehicle for 9 days, 3 T3-L1 cells were homogenized and processed in buffer composed in mM by: 50 HEPES, 40 NaCl, 50 NaF, 2 EDTA, 10 sodium pyrophosphate, 10 sodium glycerophosphate, 2 sodium orthovanadate, and 1% Triton-X100 and EDTA-free protease inhibitors. Identical amounts of protein aliquots from 3 T3-L1 cell lysates were resolved on Nupage gradient gels (4–12%, Invitrogen Life Technologies) and transferred to nitrocellulose membranes. After blockage with 5% milk for 1 h, membranes were overnight incubated at 4 °C with the following primary antibodies: mitochondrial complex I subunit NDUFB8 (20 kDa), complex II subunit 30 kDA (30 kDa), complex III subunit Core 2 (48 kDa), COXIV subunit I (40 kDa), complex V ATP synthase subunit alpha (53 kDa) (OXPHOS kit, MitoSciences, Inc.) and gamma-tubulin (~ 50 kDa #5886, Cell Signaling, Beverly, MA, USA) in 5% milk (1:1000). After washing, membranes were subsequently incubated with appropriate peroxidase-conjugated secondary antibody (1:5000) for 1 h and developed using the ECL enhanced chemiluminescence substrate (GE Healthcare Life Sciences, Björkgatan, Uppsala). Densitometric analyses were performed using the ImageJ software (National Institutes of Health, Bethesda, MD).

### RNA extraction, reverse transcription and quantitative real-time PCR (real-time qRT-PCR)

Total RNA from 3 T3-L1 cell lysates was extracted using Trizol (Invitrogen Life Technologies), analyzed for quality on agarose gel and absorbance ratios of 260/280 nm and 260/230 nm, and reverse transcribed to cDNA using the SuperScript III cDNA kit (Invitrogen Life Technologies). Gene expression was evaluated by real-time qRT-PCR using a Rotor Gene (Qiagen, Roermond, Netherlands) and SYBR Green as fluorescent dye (Qiagen) with 36B4/Rplp0 as housekeeping gene. The reaction conditions were as follows: 95 °C for 5 min, then 40 cycles of 95 °C for 5 s and 60 °C for 10 s. PCR products were run on agarose gel to confirm the size of the fragment and specificity of amplification. Primers used were: *Pnpla2* (5′-3′ sense: GGTCCTCTGCATCCCTCCTT; 5′-3’antisense: CTGTCCTGAGGGAGATGTC), *aP2* (5′-3′ sense: AAGGTGAAG AGCATCATAACCCT; 5′-3’antisense: TCACGCCTTTCATAACACATTCC) and *36B4* (5′-3′ sense: TAAAGACTGGAGACAAGGTG; 5′-3’antisense: GTGTACTCAGTCTCCAC AGA). Data were obtained as ct values (ct = cycle number at which logarithmic PCR plots cross a calculated threshold line) and used to determine ∆ct values (∆ct = (ct of the target gene) - (ct of the housekeeping gene). Data were expressed as arbitrary units using the following calculation: [expression = 1000×(2^-Δct^) arbitrary units (AU)].

### Mitochondrial mass determination

The methodology was based on the protocol used by Shen et al. [25]. A fluorescent probe (Mito-Tracker Green FM; Molecular Probes, Eugene, OR, USA) was used to determine the mitochondrial mass of adipocytes [27]. Mature adipocytes (9 days of differentiation) were treated with palmitic or palmitoleic acid, trypsinised and centrifuged at 1500×g, 4 °C, for 5 min, resuspended in KRH buffer containing 0.1% BSA (*w*/*v*) and then incubated with 0.1 μmol/L MitoTracker Green FM in KRH buffer for 30 min at 37 °C. Cells were centrifuged at 1500×g, 4 °C, for 5 min and resuspended in 400 μL of fresh KRH buffer. Fluorescence measurements were carried out in CALIBUR cytometer (BD) in the FL1 channel. Ten thousand events were analyzed per experiment. Data were analyzed using Cell Quest software.

### Citrate synthase activity assay

Citrate synthase (EC 4.1.3.7) maximum activity was determined as previously described by Alp et al. [28]. Briefly, after 9 days treatment with FA or vehicle, 3 T3-L1 cells were homogenized (vortex) in extraction buffer containing Tris-HCl (50 mM), EDTA (1 mM), leupeptin (50 μM), and aprotinin (5 μM), pH 7.4, and centrifuged (16,000 *g*, 30 s, 4 °C). The assay buffer consisted of Tris HCl (100 mM), DTNB (0.2 mM), acetyl-CoA (100 mM), and Triton (1%), pH 6.5. The reaction was started by adding 10 uL of oxaloacetic acid (500 mM) 25 °C. The absorbance was monitored for 10 min at 420 nm. Protein concentration in the supernatants was determined using a BCA® protein assay kit (PIERCE Biotechnology, Rockford, IL). The maximal enzyme activity was expressed as micromoles per minute per microgram of protein.

### Statistical analysis

Results are expressed as mean ± SEM. One-Way ANOVA followed by Tukey post-hoc test were used to compare the effects of different treatments. Analysis was performed using GraphPad Prism 5.0 software (GraphPad Software, Inc., San Diego, CA, USA). The level of significance was set at *p* ≤ 0.05.

## Results

**Fig. 1:**
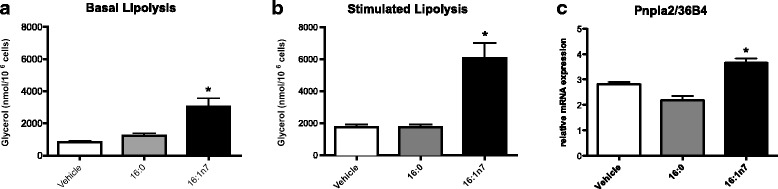
Basal (**a**) and isoproterenol-stimulated (**b**) lipolysis measuring by release of glycerol (nanomoles per 10^6^ cells). **c** mRNA levels of *Pnpla2* gene (arbitrary units). Experiments performed in differentiated 3 T3-L1 adipocytes treated for 9 days with vehicle, palmitic acid (16:0, 100 μM) or palmitoleic acid (16:1n7, 100 μM). Results are means ± SEM. * *P* < 0.05 16:1n7 vs. all groups. The results are the average of 3 independent experiments (n = 4/experiment)

First, we examined the effect of 9-day treatment with palmitoleic or palmitic acid on 3 T3-L1 adipocyte lipolysis. Palmitoleic acid increased both basal (by 3-fold) and stimulated (by 3.5-fold) lipolysis as measured by the glycerol released to the medium [mean ± SEM, Basal (vehicle: 828.9 ± 95.4; 16:1n7: 3027.2 ± 511) and stimulated (vehicle: 1760.3 ± 174; 16:1n7: 6077.7 ± 822) nMol/10^6^ cells, *p* < 0.05] (Fig. 1a and b, respectively). *Pnpla2* mRNA levels were significantly increased (by 30%) in cells treated with palmitoleic acid (Fig. 1c). We further investigated whether 16:1n7 would also promote an increase in free fatty acid (FFA) release by these cells (dictated by a glycerol/FA proportion of 1:3, as products of TAG hydrolysis). Palmitoleic acid did not cause significant increase of FFA release into the medium, that is, no difference was observed among the groups. Interestingly, this profile was slightly altered by pretreatment with etomoxir (a potent inhibitor of fatty acid mitochondrial uptake and oxidation), since 16:1n7 + etomoxir treated cells presented an increase by 5% in the FFA release into the medium (mean ± SEM, 16:1n7: 59.2 ± 0.8; 16:1n7 + etomoxir: 63.3 ± 0.44 μMol/L, *p* < 0.05) (Fig. 3b). We did not observe any effect of palmitic acid on lipolytic activity.

**Fig. 2:**
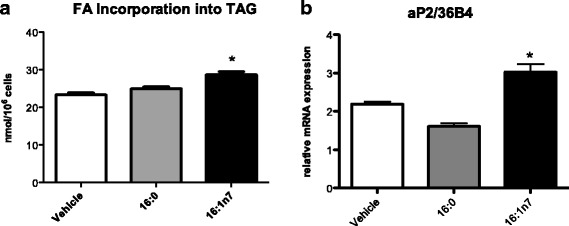
**a** [1-^14^C]-palmitate incorporation into TAG (nanomoles of incorporated [1-^14^C]-palmitate per 10^6^ cells) and **b** mRNA levels of *aP2* (arbitrary units). Experiments performed in differentiated 3 T3-L1 adipocytes treated for 9 days with vehicle, palmitic acid (16:0, 100 μM) or palmitoleic acid (16:1n7, 100 μM). Results are means ± SEM. * *P* < 0.05 16:1n7 vs. all groups. The results are the average of 3 independent experiments (*n* = 6/experiment)

We hypothesized that palmitoleic acid should boost TAG lipolysis/FA re-esterification cycle. To test this hypothesis, we analyzed if FA esterification was raised in cells treated with palmitoleic acid. Indeed, an increase of [1-^14^C]-palmitate incorporation into TAG (by 40%) was observed in cells treated with palmitoleic acid (mean ± SEM, vehicle: 23.42 ± 0.5; 16:1n7: 28.73 ± 0.8 nMol/10^6^ cells, *p* < 0.05) (Fig. 2a). Moreover, *aP2* mRNA levels were positively regulated by palmitoleic acid (Fig. 2b). These results show that FA incorporation into TAG is increased in 3 T3-L1 adipocytes treated with palmitoleic acid. No effect on the synthesis of TAG was observed in cells treated with palmitic acid.

**Fig. 3:**
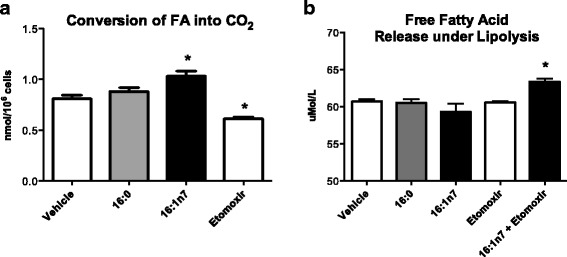
**a** Conversion of [1-^14^C]-palmitate into CO_2_ (nanomoles of converted [1-^14^C]-palmitate into CO_2_ per 10^6^ cells) and **b** Free fatty acids released under lipolysis (μMol per liter per well). Experiments performed in 3 T3-L1 adipocytes under 9-days treatment with vehicle, etomoxir (40 μM), palmitic acid (16:0, 100 μM) or palmitoleic acid (16:1n7, 100 μM). Results are means ± SEM. * *P* < 0.05 vs. all groups. The results are the average of 3 independent experiments (*n* = 4/experiment)

Next, we investigated if palmitoleic acid could enhance FA beta-oxidation. The conversion of [^14^C]-palmitate to CO_2_ was raised by 30% in cells treated with palmitoleic acid when compared to control cells (mean ± SEM, vehicle: 0.8 ± 0.033; 16:1n7: 1.03 ± 0.05 nMol/10^6^ cells, *p* < 0.05) (Fig. 3a). No change in FAO was observed in cells treated with palmitic acid when compared to control. Disruption of fatty acid oxidation by etomoxir in a dose of 40 μM was confirmed in this experiment by partial attenuation (~ 25%) of fatty acid oxidation.

**Fig. 4:**
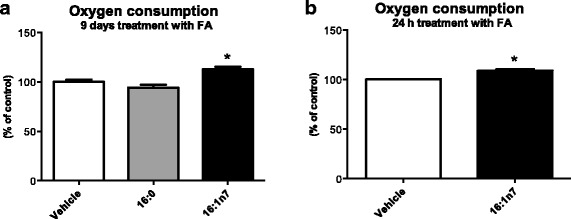
Oxygen consumption by 3 T3-L1 adipocytes under (**a**) chronic (9 days) and **b** acute (24 h) treatments with vehicle, palmitic acid (16:0, 100 μM, respectively) or palmitoleic acid (16:1n7, 200 or 100 μM, respectively). Results expressed as percentage of control. Results are presented as means ± SEM. * *P* < 0.05 16:1n7 vs. all groups. The results are the average of 3 independent experiments (*n* = 6/experiment)

Other mitochondrial parameters, such as ATP levels and oxygen consumption, were also measured in 3 T3-L1 cells. FA oxidation is a metabolic pathway that requires oxygen to take place. We performed oxygen consumption measurements in 3 T3-L1 adipocytes treated with fatty acids using a modular system for high-resolution respirometry (HRR), the Oroboros Oxygrapk-2 k (O2k). Oxygen consumption was raised in cells treated with palmitoleic acid for 9 days (by 13%, Fig. 4a) and 24 h (by 10%, Fig. 4b). So, palmitoleic acid stimulates basal energetic metabolism in white adipocytes. Palmitic acid treatment did not affect oxygen consumption in any of the conditions studied.

**Fig. 5:**
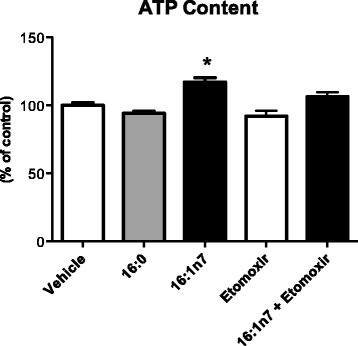
ATP content on 3 T3-L1 adipocytes under 9-day treatment with vehicle, etomoxir (40 μM), palmitic acid (16:0, 100 μM) or palmitoleic acid (16:1n7, 100 μM). Results expressed as percentage of control. Results are means ± SEM. * *P* < 0.05 16:1n7 vs. all groups. The results are the average of 3 independent experiments (*n* = 4/experiment)

We further investigated the palmitoleic effects on ATP content. 16:1n7, but not 16:0 treatment of the cells, raised ATP levels by 17% (mean ± SEM, vehicle: 100 ± 1.72; 16:1n7: 119.8 ± 1.51 percentage of control, *p* < 0.05) (Fig. 5). We hypothesized that raised FAO, secondary to raised lipolysis promoted by 16:1n7, elevates ATP production (corroborating oxygen consumption) in a magnitude capable of overcome its consumption (raised by the exacerbation of TAG/FA cycle). This hypothesis is supported here, by the experiments performed under pretreatment of the cells with etomoxir, that completely prevented the increment on ATP content induced by palmitoleic acid (Fig. 5).

**Fig. 6:**
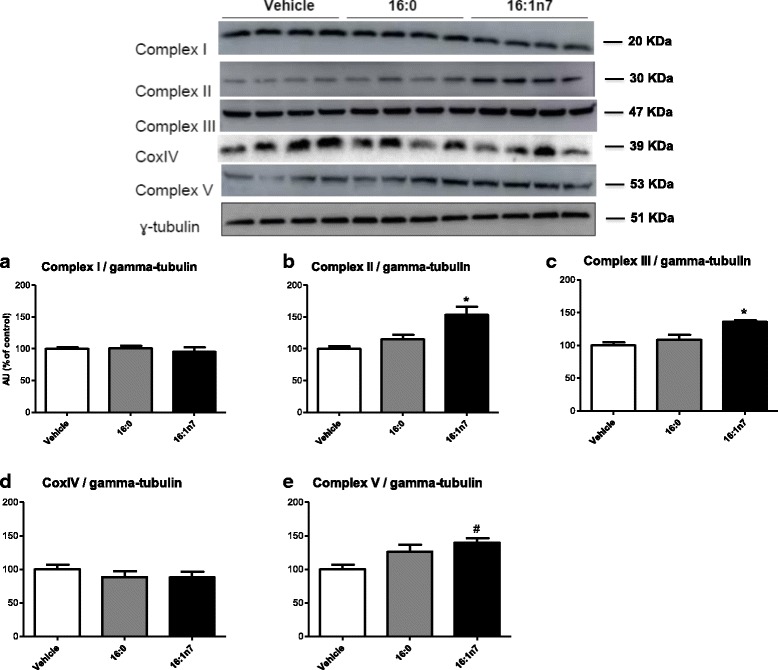
Total content of subunits representing OXPHOS proteins complexes analyzed by western blot. **a** Complex I. **b** Complex II. **c** Complex III. **d** CoxIV. **e** Complex V. Experiments were performed in differentiated 3 T3-L1 adipocytes treated for 9 days with vehicle, palmitic acid (16:0, 100 μM) or palmitoleic acid (16:1n7, 100 μM). Results expressed as arbitrary units. Results are presented as means ± SEM. * *P* < 0.05 16:1n7 vs. all groups, # *P* < 0.05 16:1n7 vs. vehicle. The results are the average of 3 independent experiments (*n* = 4/experiment)

**Fig. 7:**
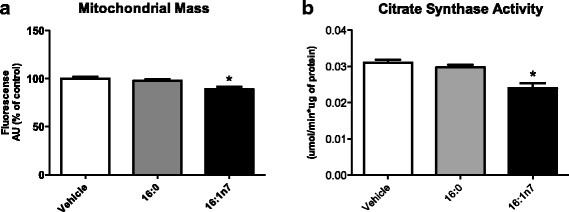
**a** Mitochondrial mass (percentage of florescence). **b** Citrate synthase activity (micromoles per minute per microgram of protein). Experiments performed in differentiated 3 T3-L1 adipocytes treated for 9 days with vehicle, palmitic acid (16:0, 100 μM) or palmitoleic acid (16:1n7, 100 μM). Results are means ± SEM. * *P* < 0.05 16:1n7 vs. all groups. The results are the average of 3–4 independent experiments (*n* = 6/experiment)

Since the augmented ATP concentration observed in our studies suggest an increment in mitochondrial OXPHOS efficiency, we next evaluated protein expression of subunits representing complex II, III, and V of the mitochondrial electron transport chain. Complex II and III were positively modulated by palmitoleic acid (by 54% and by 36%, respectively), but not by palmitic acid, when compared with the control group (Fig. 6b and c). Likewise, complex V was positively modulated by palmitoleic acid (by 40%) when compared to the control group (Fig. 7e). However, palmitoleic acid did not elicit any change in the content of mitochondrial complexes I and IV (Fig. 6a and d, respectively).

Mitochondria mass was evaluated by flow cytometry, according to the methodology proposed by Shen and colleagues [25]. A decrease of ~ 10% in mitochondria mass was observed in cells treated with palmitoleic acid. There was no effect of palmitic acid on this parameter (Fig. 7a). Another parameter to measure mitochondria content is citrate synthase activity. Palmitoleic acid (but not palmitic acid) led also to a reduction of ~ 10% in citrate synthase activity (mean ± SEM, vehicle: 0.031 ± 0.0005; 16:1n7: 0.024 ± 0.0008 μMol/min/μg of protein, *p* < 0.05) (Fig. 7b), when compared with the control group.

## Discussion

3 T3-L1 adipocytes were treated with palmitic acid or palmitoleic acid, being the latter recently described to induce important effects on carbohydrate and lipid metabolism in liver and skeletal muscle [12, 15, 29, 30]. We have previously demonstrated that palmitoleic acid controls important metabolic processes also in adipose tissue. We reported increased lipolysis and glucose uptake, together with changes in expression of related genes and proteins (ATGL, HSL, AMPK and GLUT4) in white adipocytes [18, 19]. In the present work, we observed that palmitoleic acid modulates other aspects of adipocyte metabolism, mainly mitochondria bioenergetics. Palmitoleic acid (but not palmitic acid) enhanced energy expenditure in adipocytes through TAG/FA cycle stimulation, FA oxidation, oxygen consumption and increased protein expression of subunits representing complex II, III, and V of the mitochondrial electron transport chain. The treatment of the adipocytes with palmitic acid (16:0), different from palmitoleic (16:1n7), showed no marked effect on the parameters herein studied.

An increase in both basal and isoproterenol-stimulated lipolysis, together with augmented gene expression of the *Pnpla2*, were observed in 3 T3-L1 cells treated with palmitoleic acid (9 days, from the induction of preadipocyte differentiation) when compared to the palmitic acid and control groups. These findings corroborate previous ex vivo results obtained by our group [18]. We have shown that there is an increase of lipolysis in primary adipocytes extracted from epididymal fat pads of mice treated with palmitoleic acid (by gavage) for 10 days and also, in mature (6 days post-differentiation) 3 T3-L1 cells treated with palmitoleic acid (by 24 h), via a PPARα-dependent mechanism. The increment in lipolysis promoted by palmitoleic acid was estimated according to the concentration of glycerol released into the medium. A concomitant increment of FFA released from adipocytes [dictated by a glycerol/FA proportion of 1:3, as products of TAG hydrolysis] treated with palmitoleic acid was not observed. This suggests that the fatty acids originated from lipolysis of cells treated with palmitoleic acid could be used in other metabolic pathways such as: (1) re-esterification, (2) mitochondrial oxidation or (3) mitochondrial uncoupling, including the possibility of overlapping pathways. We ruled out mitochondrial uncoupling due to the fact that cellular ATP levels were augmented upon palmitoleic acid treatment (Fig. 5) and UCP1/2 mRNA levels are not altered under this condition (data not shown).

To test the hypothesis 1, we analyzed if FA esterification was increased in cells treated with palmitoleic acid for 9 days. Palmitoleic acid did increase [1-^14^C]-palmitate incorporation into TAG as well as *aP2* mRNA levels in adipocytes. *aP2* gene encodes a protein involved in FA uptake and transport to be then associated to coenzyme A (a reaction catalyzed by acyl-CoA synthetase) to generate acyl-CoA. Corroborating these findings, we demonstrated an increase in the generation of glycerol 3-phosphate (G3-P) via glycolysis (required for esterification of fatty acids to TAG), as evidenced by the higher rates of glucose incorporation into the glycerol fraction of TAG [18]. In another recent work published by our group [19] an increase of glucose uptake by adipocytes, via a mechanism that involves AMPK, promoted by palmitoleic acid was demonstrated. This observation is compatible with increased G3-P formation from glucose, since there was not seen any increase in gene expression of the glyconeogenic regulatory enzyme PEPCK or in glycerol kinase activity in this study, the two other possible sources of G3-P [31]. These results suggest that palmitoleic acid promotes FA re-esterification and TAG/FA futile cycle stimulation.

A concomitant increase in lipolysis and FA re-esterification implies in stimulation of the substrate cycle (or “futile” cycle) TAG/FA cycle [32, 33]. In mammals, an increase in this cycle activity together with raised fatty acid oxidation can lead to augmented basal metabolic rate (BMR) and total energy expenditure [34] as well as greater sensitivity to hormonal control of energy metabolism (e.g. lipolytic and lipogenic activities). Additionally, the stimulation of this cycle is a mechanism associated with induction of thermogenesis during cold exposure [35] and also when plasma leptin levels are high [32].

In the next step, we investigated if palmitoleic acid could enhance FA beta-oxidation and oxygen consumption. Maassen, Romijn and Heine [36] suggested that FA oxidation in adipocytes might also contribute to the retention of FA in these cells, thus avoiding excessive release of FA into the circulation when lipolysis is stimulated reducing lipotoxicity. We observed an increase (by 30%) in FA oxidation in 3 T3-L1 adipocytes treated with palmitoleic acid. This effect maybe underestimated due to the fact that palmitoleic acid can compete with labeled [1-^14^C]-palmitate (50uM) added to the medium during this test for the beta-oxidation pathway.

Despite the fact that some studies describe FA oxidation as a minor metabolic pathway in white adipocytes, especially when compared to FA re-esterification [37–39], Dib, Bugge and Collins [40] claim that FA oxidation in WAT is not negligible and contributes from 5 to 10% of the BMR in lean and obese adults, respectively. Noteworthy, the percentage of endogenous FA oxidized in primary rat white adipocyte was calculated and found to be around 0.2%, what seems small when compared to re-esterified (~ 50%) and released endogenous FFA (~ 50,1%) [41]. We estimated herein the amount of FFA released by the cells, which were around 40 nmoles/10^6^ cells. The estimated FFA released is much lower than the amount of glycerol released (by 70-fold) but it is much higher than the amount of CO_2_ generated (~ 1 nmol/10^6^ cells). This finding corroborates the data that FFA re-esterification and endogenously released FFA are the major pathways when compared to FFA oxidation. Anyway, pretreatment with etomoxir, that has been shown to bind CPT-1 with high affinity, thereby preventing mitochondrial uptake of fatty acids [42], caused an increment in the FFA release into the medium by the cells treated with palmitoleic acid. The disruption of fatty acid oxidation by etomoxir was confirmed here, by the attenuation of fatty acid oxidation.

Wang and cols [41] claim that FFA oxidation assays are usually performed during a short period of time (a 2-h period or so) and, if extrapolated to years, this oxidative pathway could reduce more than 1 kg of fat mass per 50 kg of fat mass per year in humans depending on if there is combined fasting or not. Therefore, WAT FAO might be not so negligible as previously thought to be. Actually, efficient mitochondrial WAT FAO may increase respiratory capacity, reduce adipocyte size, enhance lipolysis and reduce lipotoxicity [6, 38, 43]. A recent study with human twins showed that adiposity is correlated with downshifting of FAO and that, indeed, mitochondrial biogenesis, oxidative metabolic pathways and OXPHOS proteins in subcutaneous adipose tissue are downregulated in acquired obesity [44]. Altogether these studies corroborate the hypothesis that mitochondrial WAT FAO stimulation has relevant effects on body energy balance.

Together with increased FAO, an increment in basal O_2_ consumption was observed in 3 T3-L1 cells treated with palmitoleic acid, which suggests an increase in BMR of these cells. This enhancement in oxygen consumption could be explained by an increase in FAO, mitochondrial uncoupling or both [45].

The augmentation in O_2_ consumption observed in the present study supports the consistent raise in FAO catabolic pathway. In addition, cellular ATP levels were raised by 17% as well as protein levels of the subunit ATP-5A of the OXPHOS complex V (ATP synthase, Fig. 6e) upon palmitoleic acid treatment. Pretreatment with etomoxir completely prevented this effect, demonstrating the dependent effect of FAO increase for the palmitoleic effects here observed. Altogether, the raise in all these mitochondrial parameters: oxygen consumption, FAO and ATP generation, points out to enhanced mitochondrial oxidative phosphorylation efficiency [46] upon palmitoleic acid treatment. Although the palmitoleic acid effects seems mild, according Gao and colleagues [47] mitochondria do not require major changes to reflect a prominent metabolic effect. In contrast, abrupt mitochondrial changes are toxic and induce cell death. This work showed that both sudden increases or decreases cause mitochondrial damage by high levels of glucose and free fatty acids in 3 T3-L1 adipocytes.

At first, palmitoleic acid-induced increase in cellular ATP levels seems odd since raised AMP/ATP ratio is the main factor that leads to AMPK phosphorylation and activation. In addition, given the crucial role of the acyl-CoA synthetase in the re-esterification of FA released from lipolysis [48], this finding looks indeed unexpected. Acyl-CoA synthetase catalyzes the activation of 1 FA in 1 acyl-CoA, consuming 1 ATP (1 AMP and pyrophosphate). In fact, it utilizes two equivalents of ATP since pyrophosphate is cleaved into 2 inorganic phosphate molecules, breaking a high-energy phosphate bond. Nevertheless, FAO generates NADH and FADH_2_, which enter the electron transport chain to produce ATP. Our hypothesis is that raised FAO (secondary to raised lipolysis) promoted by 16:1n7, elevates ATP production (corroborating oxygen consumption increase) in a magnitude capable of overcome its consumption (raised by the exacerbation of TAG/FA cycle). This hypothesis is supported here, by the experiments performed under pretreatment of the cells with etomoxir, that completely prevented the increment on ATP content induced by palmitoleic acid. Actually, Baldwin et al. [49] calculated that the energy required for TAG/FA cycle is around 8 molecules of ATP per release and re-esterification of 3 molecules of FA (2,7 ATP/FA). This energetic demand could be provided by mitochondria FAO (fasting) and glucose oxidation (fed state) [50]. Indeed, the effectiveness of fuel oxidation by mitochondrial relies on mitochondria content, mitochondrial activity and mitochondrial efficiency to synthesize ATP from the oxidation of substrates [46]. Noteworthy, relative to their dry mass, FA provide twice as much ATP as carbohydrates: six times more when comparing stored FA to stored glycogen [51]. It is importante to emphasize that cell lines in culture, including 3 T3-L1 cells, display a high rate of glycolysis. However, we can assume herein that glycolysis is not responsible for the increament in ATP levels, since the results showed that when CPT1 was inhibited in cells treated with palmitoleic acid, the ATP levels increase was completely abolished.

The augmented ATP concentration observed corroborates our data of an increment in mitochondrial OXPHOS efficiency (increased protein expression of subunits representing OXPHOS complex II, III and IV) in cells treated with 16:1n7. Raised ATP consumption is observed when lipolysis occurs simultaneously with FA re-esterification (TAG/FA cycle) [52]. As a paradox, augmented basal and isoproterenol stimulated lipolysis (promoted by 16:1n7) increases AMP/ATP ratio, which is known to promote AMPK activation [53], that, in turn, would stimulate catabolic pathways in order to increase ATP production, corroborating the data of raised beta-oxidation and oxygen consumption demonstrated herein. These findings also support the previous data shown by our group that 16:1n7 led to an increase on glucose uptake and oxidation associated with AMPK activation [19].

WAT mitochondria are potential targets in the search for the development of therapies to prevent and treat obesity, inflammation, insulin resistance and related disorders [6, 40, 54]. Ahmadian et al. (56) suggested that augmented lipolysis could result in a change in adipocyte metabolism via greater use of FA and enhanced energy expenditure, protecting against obesity [55]. The fact that palmitoleic acid reduced citrate synthase activity corroborates the results of decreased mitochondrial mass under the effect of this fatty acid, suggesting more efficient mitochondria, although fewer in number, in these adipocytes treated with palmitoleic acid. Indeed, relevant metabolic parameters of these cells were increased, such as oxygen consumption, beta-oxidation, lipolysis and lipogenesis, which corroborates an increased intrinsic mitochondrial function as a physiologic adaptation. It is currently accepted that mitochondria are dynamic organelles, which adjust according to the cell’s energy demand.

Mitochondrial function (particularly OXPHOS uncoupling and FA oxidation) is considered a crucial factor to improve systemic insulin sensitivity [56–59]. Mitochondrial oxidative capacity in WAT correlates negatively with adiposity [50]. For example, a WAT knockout mouse for Liver Receptor X alpha (ATaKO mouse) was generated and presented more weight gain and fat mass on a high-fat diet compared with wild-type controls as a result of the decrease in WAT lipolytic and oxidative capacities [60]. Accordingly, LXR activation in vivo and in vitro led to decreased WAT adipocyte size and increased glycerol release from primary adipocytes, respectively, with a concomitant increase in oxygen consumption [40]. Thereby, novel compounds with potential to stimulate lipolysis, TAG/FA cycle, FA oxidation in WAT are desired in therapies focusing obesity and related diseases.

## Conclusions

**Fig. 8:**
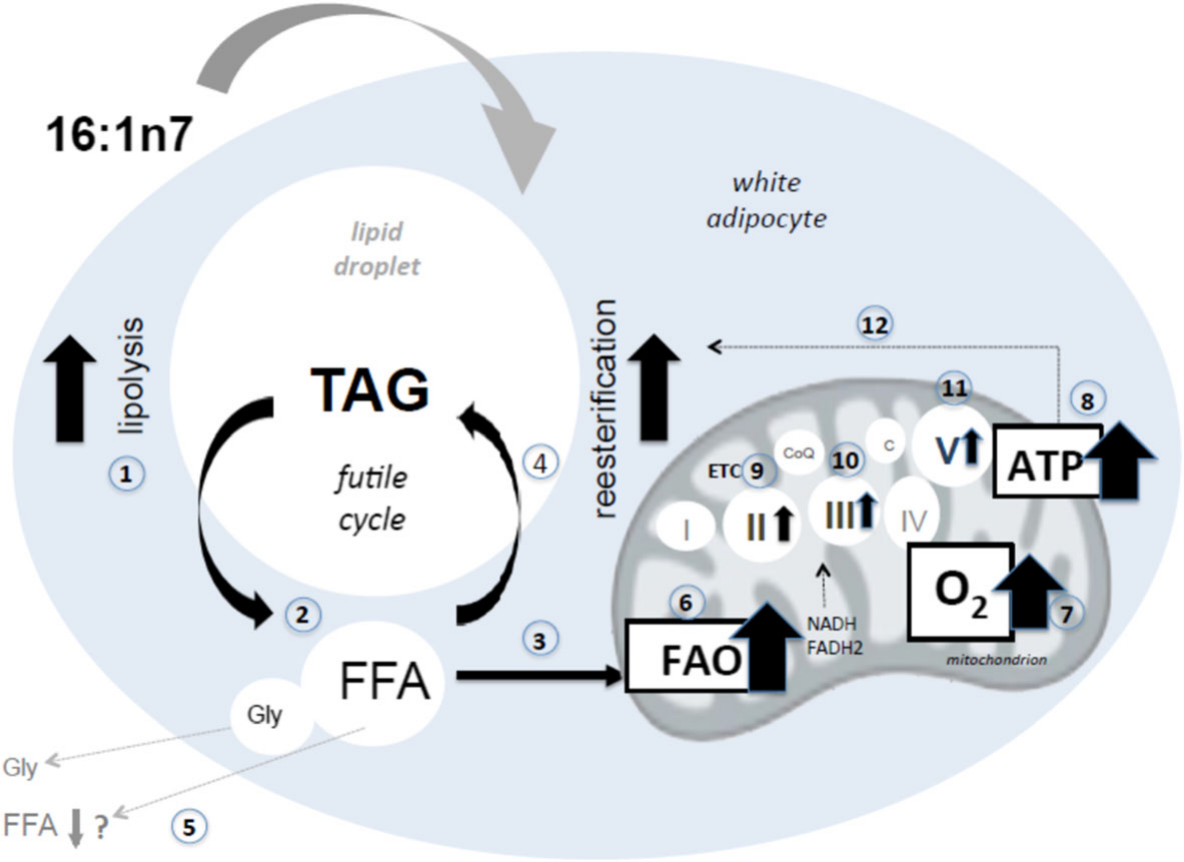
Treatment with 16:1n7 for 9 days stimulated both lipolysis (1) and FFA re-esterification (4) in 3 T3-L1 adipocytes. Raised lipolysis leads to FFA (2) and glycerol release. FFA follow different fates (3): re-esterification into TAG (4), plasma (5) and mitochondria oxidation (6). 16:1n7 raised mitochondrial parameters such as FAO (6), oxygen consumption (7) and ATP generation (8). In addition, ETC protein expression is also elevated by 16:n7: complex II (9), complex III (10) and ATP synthase (11). ATP augmentation contributes to reesterification (12). Altogether, 16:1n7 enhances WAT futile cycle (lipolysis-re-esterification cycle) as well as mitochondria bioenergetics

In conclusion, our data show that palmitoleic acid plays an important role on WAT metabolic and mitochondrial function, suggesting an increase in energy expenditure of adipocytes by TAG/FA cycle acceleration, FA oxidation and oxygen consumption and, thus, raised cell energy metabolism. We presented herein evidence indicating that palmitoleic acid, by concerted action on lipolysis, mitochondrial FA oxidation, ATP content and oxygen consumption, contributes to enhance white adipocyte energy expenditure and could be considered as a possible candidate for clinical research related to adipose tissue (Fig. 8). We believe that palmitoleic acid treatment alone or its combination with caloric restriction and/or exercise is a promise for future obesity treatment/prevention. Nevertheless, the mechanisms involved in palmitoleic-induced TAG/FA cycle stimulation via mitochondria function have to be fully elucidated.

## Abbreviations

AMPK: AMP-activated protein kinase
aP2/FABP4: Fatty acid binding protein 4
ATGL/PNPLA2: Adipocyte triacylglycerol lipase
ATP: Adenosine 5′-triphosphate
BMR: Basal metabolic rate
CCCP: Carbonyl cyanide m-chlorophenyl hydrazine
COXIV: Cytochrome c oxidase subunit IV
DMEM: Dulbeccos modified eagle medium
ETC: Electron tranport chain
FA: Fatty acid
FADH_2_: Flavin adenine dinucleotide;
FAO: Fatty acid oxidation
FAS/FASN: Fatty acid synthase
FBS: Fetal bovine serum
FFA: Free fatty acid
G3-P: Glycerol 3-phosphate
G6PDH/H6PDH: Glucose 6 phosphate dehydrogenase
GLUT: Glucose transporter
HRR: High resolution respirometry
HSL/LIPE: Hormone sensitive lipase
KRH: Krebs ringer hepes bicarbonate buffer
LXRA: Liver X receptor alpha
NADH/NAD +: Nicotinamide adenine dinucleotide
OXPHOS: Oxidative phosphorylation
PBS: Phosphate buffered saline
PEPCK: Phosphoenolpyruvate carboxykinase
PKB or Akt: Protein kinase B
PPARα: Peroxisome proliferator-activated receptor alpha
TAG: Triacylglycerol
TAG/FA cycle: Triacylglycerol/fatty acid cycle
T2DM: Type 2 diabetes mellitus
UCP: Uncoupling protein
WAT: White adipose tissue
